# Two novel *GJA1* variants in oculodentodigital dysplasia

**DOI:** 10.1002/mgg3.882

**Published:** 2019-07-25

**Authors:** Nikolai P. Pace, Valerie Benoit, David Agius, Maria Angela Grima, Raymond Parascandalo, Pascale Hilbert, Isabella Borg

**Affiliations:** ^1^ Centre for Molecular Medicine and Biobanking University of Malta Msida Malta; ^2^ Département de Biologie Moléculaire Institut de Pathologie et de Génétique ASBL Gosselies Belgium; ^3^ Department of Ophthalmology Mater Dei Hospital Msida Malta; ^4^ Department of Medicine Mater Dei Hospital Msida Malta; ^5^ Department of Pediatrics Mater Dei Hospital Msida Malta; ^6^ Department of Pathology, Faculty of Medicine and Surgery University of Malta Msida Malta; ^7^ Medical Genetics Unit, Department of Pathology Mater Dei Hospital Msida Malta

**Keywords:** connexin 43, *GJA1* gene, oculodentodigital dysplasia

## Abstract

**Background:**

Oculodentodigital dysplasia (ODDD) is a rare disorder with pleiotropic effects involving multiple body systems, caused by mutations in the gap junction protein alpha 1 (*GJA1*) gene. *GJA1* gene encodes a polytopic connexin membrane protein, Cx43, that is a component of connexon membrane channels.

**Methods:**

We describe two unrelated female probands referred for a genetic review in view of a dysmorphic clinical phenotype.

**Results:**

Two novel missense mutations in *GJA1* that substitute conserved amino acids in the first and second transmembrane domains (NM_000165.5: c.77T>C p.Leu26Pro and NM_000165.5:c.287T>G p.Val96Gly) were detected through targeted sequencing of *GJA1*. These variants were detected in the heterozygous state in the two Maltese probands and segregated with the disease phenotype.

**Conclusion:**

This report further expands the mutational spectrum of ODDD.

## 1 INTRODUCTION

Oculodentodigital dysplasia (ODDD) (OMIM#164200) is a rare disorder characterized by various ophthalmic, dental, skeletal, and craniofacial anomalies. It has a high penetrance but its phenotypic expression is variable (Judisch, Martin‐Casals, Hanson, & Olin, 1979). ODDD is primarily an autosomal dominant disorder caused by mutations in the gap junction protein alpha 1 (*GJA1*, OMIM#121014, HGNC ID: 4274, NM_000165.5) gene, which encodes the connexin 43 (Cx43) transmembrane protein (Paznekas et al., 2003).

The phenotypic features characteristic of ODDD are well described (Hennekam, Allanson, & Krantz, 2010). Typical ophthalmologic abnormalities include microphthalmia, microcornea, and various anomalies that involve the iris, which can lead to glaucoma, cataracts, and optic neuropathy. Dental features involve both the primary and secondary dentition and include enamel hypoplasia, microdontia, hypodontia, and premature loss of teeth. Typical skeletal anomalies include bilateral syndactyly of the 2nd to 4th toes and/or of the 4th and 5th fingers, camptodactyly and clinodactyly. Affected individuals also exhibit craniofacial dysmorphic features, including a thin nose, narrow anteverted nares, short palpebral fissures, and microcephaly. A variety of neurological manifestations have been reported, including lower limb spastic paraparesis, ataxic gait, seizures, neurogenic bladder, and uncommonly, psychomotor retardation. These neurological features of ODDD involve 30% of cases (De Bock, Kerrebrouck, Wang, & Leybaert, 2013). ODDD exhibits extensive phenotypic pleiotropy, and abnormalities involving the heart, lymphatics, and skin have also been reported (Brice et al., 2013; Kogame et al., 2014; Paznekas et al., 2003).

Connexin 43 (Cx43) is a ubiquitously expressed protein that forms hexameric connexin hemichannels, which interact with connexin hemichannels on apposed membranes to form gap junctions. Gap junctions play critical physiological roles, allowing intercellular exchange of ions, metabolites and second messengers (Laird, 2006). The human genome encodes 21 connexin genes, which are co‐expressed in diverse combinations to form different channels. Each connexin protein is composed of four transmembrane domains, two extracellular loops and cytoplasmic amino‐ and carboxyl termini (Yeager & Gilula, 1992). The transmembrane and extracellular domains are highly conserved. Cx43 is the most widely studied connexin, in view of its widespread expression in most body tissues and its extensive interactions with other proteins.

A wide array of human pathologies are caused by mutations in connexin genes, including peripheral neuropathies, skin disorders, non‐syndromic hearing loss and congenital cataract (Abrams & Scherer, 2012; Krutovskikh & Yamasaki, 2000; Lee & White, 2009). Mutations in *GJA1* lead to an array of developmental abnormalities in ODDD. The mutational spectrum in *GJA1* has been extensively reviewed (Laird, 2014). Most changes are dominant missense mutations that alter the sequence of the first two‐thirds of the Cx43 protein. Additionally, dominant frameshift, deletion, and duplication variants have also been described, as well as two autosomal recessive variants. The functional effect of a number of *GJA1* variants has been evaluated using in vitro cell models, and these studies have shown that even slight sequence changes in Cx43 can compromise gap junction function (Shao et al., 2012).

In this report, we describe two unrelated patients with clinical features of ODDD. Sanger sequencing of *GJA1* gene in the probands and family members identified two novel variants that segregate with the phenotype, suggestive of causality.

### 1.1 Ethical compliance

This study was approved by the local institutional ethics review board and written informed consent was obtained.

### 1.2 Clinical presentation

**Figure 1:**
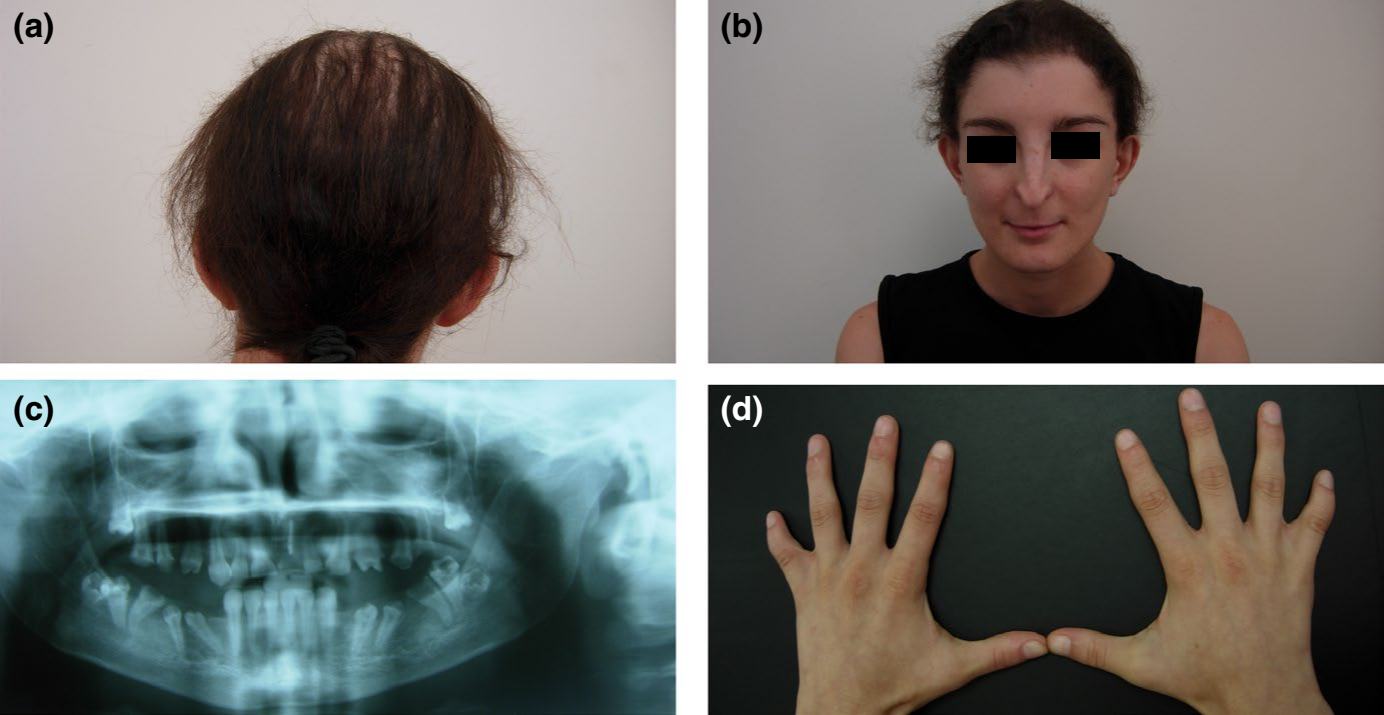
Clinical features in proband 1 with the *GJA1* NM_000165.5: c.287T>G p.Val96Gly mutation. (a) shows sparse fine lusterless hair (b) front view of face showing distinctive nose and hypoplastic alae nasi. (c) Orthopantogram showing narrow maxilla, microdontia, hypodontia, and widely spaced teeth with irregular roots (d) both hands showing fifth digit camptodactyly and syndactyly of the fourth and fifth digits

The undiagnosed probands were referred to the genetics clinic for review in view of multiple dysmorphic features that had presented at birth. Both probands are female, the offspring of healthy Maltese Caucasian non‐consanguineous parents. The respective clinical phenotypes are listed in Table 1 and selected clinical features from proband 1 are shown in Figure 1. Proband 2 was lost to clinical follow‐up.

**Table 1:** A comparison of the clinical phenotype of proband 1 and proband 2 at age 22 years and 8 years, respectively

	Proband 1 p.Val96Gly	Proband 2 p.Leu26Pro
Craniofacial features		
General		
Microcephaly	Present	Present
Hair	Thin, sparse, widow’s peak	Thin, sparse, widow’s peak
Ocular Features		
Microphthalmos	Present bilaterally	Present bilaterally
Bilateral convergent strabismus	Present	Present
Nystagmus	Absent	Present
Epiblepharon	Absent	Present
Telecanthus	Present	Absent
Hypertrophic mucosal membrane under eyelids	Present	Absent
Short palpebral fissures	Present bilaterally	Present bilaterally
Epicanthic folds	Present bilaterally	Present bilaterally
Oro‐dental features		
Enamel hypoplasia, hypodontia, abnormal 1° and 2° dentitions	Present	Present
Auricular features		
Low set and prominent pinnae	Present	Present
Nasal features		
Nasal bridge	Prominent	Normal
Thin nose with hypoplastic alae nasi	Present	Present
Columella	Prominent	Prominent
Other		
Hypoplastic maxilla	Present	Absent
Micrognathia	Present	Present
Skeletal features		
Hands and fingers		
Camptodactyly of 5th finger	Present bilaterally	Present bilaterally
Skin syndactyly of 4th and 5th fingers	Present bilaterally	Absent
Single digital crease on 5th finger	Present on left	Present on right
Hypoplastic middle phalanx of 5th finger	Present bilaterally	Normal
Finger clubbing	Present bilaterally	Absent
Transverse palmar crease	Absent	Present on right
Palmar keratosis	Absent	Present bilaterally
Toes and feet		
Syndactyly of 4th and 5th toes	Bilaterally present	Absent
Overriding 5th toe	Present on left	Absent
Foot length	Normal	Long bilaterally
Plantar keratosis	Absent	Absent
General features		
Thin skin	Present	Present
Pallor	Present	Present
Build	Slim	Normal
Body hair	Sparse axillary and pubic hair	Not applicable
Neurological manifestations		
Spastic paraparesis and ataxia	Present	Absent
Psychomotor delay	Mild	Normal

Proband 1 presented at the age of 22 years with facial dysmorphism, hair and weight loss, bone pain, and recent onset of unsteady gait and spastic paraparesis. Clinical features included the absence of subcutaneous fat, mild psychomotor delay, absence of pubic hair and hypoplastic carious teeth which required the use of dentures. She was also being investigated for amenorrhea. Proband 2 presented at the age of 8 years. Her clinical phenotype included various abnormal craniofacial and skeletal features; these included microphthalmia, a bilateral convergent squint, nystagmus, micrognathia, and bilateral fifth finger clinodactyly. She was intellectually normal and showed no neurological signs. Palmoplantar keratosis was absent in proband 1, while proband 2 had palmar keratosis at presentation. Both probands were born at term with birth weights of 2,800 grams (proband 1) and 2,520 grams (proband 2). G‐banded chromosome analyses carried out at birth had shown normal 46, XX female karyotypes.

### 1.3 Genetic analysis

In view of the characteristic clinical features of ODDD, the probands were screened for *GJA1* mutations by Sanger sequencing of PCR amplicons (Reference sequence NM_000165.5). *GJA1* consists of two exons, the first of which is untranslated, separated by an 11kb intron. The coding exon (exon 2) of *GJA1* was amplified in two overlapping fragments of 925 bp and 1,079 bp according to published protocol (Paznekas et al., 2003). The detected variants were analyzed for pathogenicity according to established guidelines from the American College of Medical Genetics/Association for Molecular Pathology (ACMG/AMP). A detailed description of the methodology is provided in the supplementary file Data S1.

## 2 RESULTS

**Figure 2:**
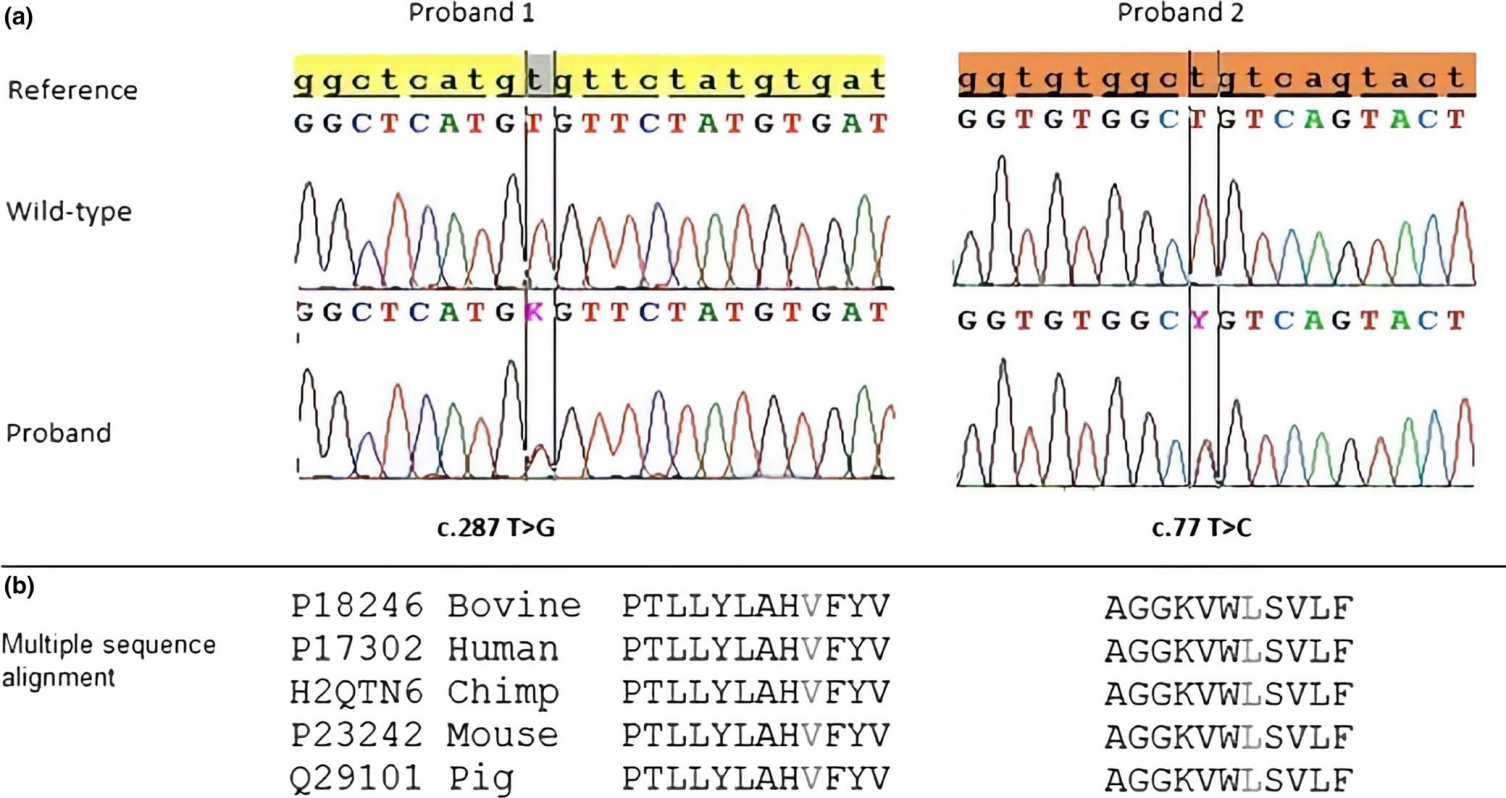
(a) Sequencing electropherograms of the *GJA1* NM_000165.5:c.77T>C p.Leu26Pro and NM_000165.5:c.287T>G p.Val96Gly variants. The top panel shows the normal wild‐type sequence in a control individual, and the bottom panel shows the heterozygous base substitutions detected in the probands. (b) Multiple sequence alignment showing conserved amino acid residues in *GJA1* flanking the substitutions detected in both probands (highlighted in red)

Nonsense mutations, frameshift mutations, in‐frame indels and variants affecting splice sites were not detected in the two probands. Two different novel missense mutations, each in the heterozygous state were detected—NM_000165.5: c.287T>G p.Val96Gly mutation in proband 1 and NM_000165.5:c.77T>C p.Leu26Pro mutation in proband 2 (Figure 2). In‐silico analysis of pathogenicity using various bioinformatics approaches is shown on Table 2 Evolutionary conservation analysis indicates that both the p.Val96Gly and p.Leu26Pro *GJA1* mutations occur at highly conserved positions within the protein, and that these residues are conserved across multiple species. Additionally, multiple lines of computational evidence provide support for deleterious effects of these missense mutations. Both mutations are absent from the gnomAD dataset and are not reported in ClinVar, Varsome and HGMD databases (Kopanos et al., 2019; Landrum et al., 2018; Stenson et al., 2017). No *GJA1* mutations were discovered in the parents of probands 1 and 2, indicating that both cases of ODDD are a product of two de‐novo and novel mutations. Furthermore, the mutations described here also lie in a well‐established functional domain of the Cx43 protein that lacks benign variation. Based on the above findings, both mutations can be considered causal for ODDD.

**Table 2:** *GJA1* mutations detected in the two probands, with in‐silico pathogenicity prediction scores and ACMG classification criteria

	Proband 1 p.Val96Gly	Proband 2 p.Leu26Pro
General prediction scores		
Position^a^	6‐121768280	6‐121768070
HGVS	NM_000165.5:c.287T>G	NM_000165.5:c.77T>C
Amino acid change	p.Val96Gly	p.Leu26Pro
DANN	0.9931	0.9991
Mutation Taster	Disease causing	Disease causing
FATHMM	Damaging	Damaging
FATHMM‐MKL	Damaging	Damaging
MetaSVM	Damaging	Damaging
MetaLR	Damaging	Damaging
SIFT	Damaging	Damaging
Provean	Damaging	Damaging
PHRED‐Scaled CADD	26.1	29.7
PolyPhen‐2^b^	Possibly damaging	Probably damaging
Evolutionary conservation scores		
GERP	5.67	6.16
LRT	Deleterious	Deleterious
Mutation Assessor	Medium	High
ACMG classification	Pathogenic according to PS2, PM1, PM2, PM5, PP2 and PP3 criteria.	Pathogenic according to PS2, PM1, PM2, PP2 and PP3 criteria.

a Position according to hg19/GRCh37 human reference genome assembly.

b PolyPhen‐2 predictions using HumVar‐trained model.

## 3 DISCUSSION

The two novel pathogenic missense mutations described in this report result in transmembrane defects of connexin 43, with the p.Leu26Pro substitution altering the first transmembrane domain and p.Val96Gly the second transmembrane domain of Cx43. Both variants segregated with the disease phenotype and were not present in the unaffected parents. Furthermore, the presence of two different mutations in the unrelated cases suggests the absence of a founder effect at this locus in the Maltese population.

Mutations in each of the four transmembrane domains of Cx43 have been described. The missense variant at position 26 (p.Leu26Pro) has not been reported previously, although a serine to proline substitution at neighboring position 27, p.Ser27Pro has been identified (Richardson, Donnai, Meire, & Dixon, 2004). Mutations that introduce proline into protein sequences are functionally unique, because the cyclical structure of its side chain confers conformational rigidity. Consequently, non‐native proline residues can disrupt secondary structures of alpha helices and beta sheets, lead to “kinks” in the protein backbone and have a destabilizing effect due to steric clash with other residues (MacArthur & Thornton, 1991). Additionally, proline lacks the capacity to form hydrogen bonds, which can effect secondary structure formation and stability (Betts & Russell, 2003).

The p.Val96Gly missense mutation identified in proband 1 has not been reported previously, although three other missense variants at this position have been described—p.Val96Ala, p.Val96Glu and p.Val96Met (Paznekas et al., 2009), (Wiest et al., 2006),(Kjaer et al., 2004). The introduction of glycine residues has functional consequences that contrast with those of proline, as its small side chain allows it to adopt a wide range of conformations compared to other residues. Consequently, mutations introducing glycine can lead to loss of stability in the hydrophobic domains of a protein.

Both missense mutations lie in highly‐conserved transmembrane domains, and in silico analysis supports their pathogenicity. Analysis using MPEx predicts that both substitutions alter the hydrophobicity of the transmembrane domain relative to the wild‐type sequence, but do not shift the residues predicted to span the cell membrane (Snider, Jayasinghe, Hristova, & White, 2009).

The mechanisms linking *GJA1* mutations to disease are complex and have been reviewed extensively (Laird, 2014). Functional studies have shown that mutations in *GJA1* can attenuate or abolish channel function, disrupt protein trafficking and modulate gap junction assembly. Critically, Cx43 transmembrane domains establish the gap junction pore and are essential for connexin localization to the plasma membrane. Furthermore, in addition to compromising channel activity, Cx43 mutations exert dominant negative effects on co‐expressed wild‐type channels, leading to a significant reduction of Cx43‐dependent gap junction intercellular transport (Roscoe et al., 2005). The diverse functional effects of *GJA1* variants are thus consistent with the pleiotropic features of ODDD. The genetic heterogeneity of the disease is similarly extensive, and several novel mutations in different ethnic groups have been recently described (Choi et al., 2018; Jamsheer et al., 2014; Porntaveetus, Srichomthong, Ohazama, Suphapeetiporn, & Shotelersuk, 2017; Tumminelli et al., 2016). Most causative mutations are missense, although nonsense and frameshift mutations have also been reported.

The phenotypic heterogeneity in ODDD is further highlighted by the varying hyperkeratotic symptoms reported here. Palmoplantar keratosis (PPK) is regarded as a minor symptom of ODDD that has been associated with C‐terminal frameshift and truncation mutations (Pfenniger, Wohlwend, & Kwak, 2011). However, PPK has also been reported in ODDD patients with *GJA1* mutations outside of the C‐terminal region (Kelly et al., 2006; Kogame et al., 2014). The presence of palmar keratosis in proband 2 harboring an N‐terminus missense mutation is thus significant as it further expands on the genotype‐phenotype associations for ODDD. Furthermore, hyperkeratosis of the palms or soles might be easily overlooked, especially if the clinical presentation is subtle. Clinicians should, therefore, be vigilant for minor cutaneous changes that are an important part of the phenotypic spectrum in ODDD.

In conclusion, this report broadens our understanding of *GJA1* variants associated with ODDD and provides the first description of this rare syndrome in individuals of Maltese ethnicity. Further in vitro studies should characterize the functional impact of these variants on connexon activity, which would lead to an improved understanding of the mechanisms leading to congenital anomalies in ODDD.

## CONFLICT OF INTEREST

The authors declare that they have no conflict of interest.

4

## Data Availability

The data that support the findings of this study are available from the corresponding author upon reasonable request.

## References

[mgg3882-bib-0001] Abrams, C. K. , & Scherer, S. S. (2012). Gap junctions in inherited human disorders of the central nervous system. Biochimica et Biophysica Acta, 1818(8), 2030–2047. 10.1016/j.bbamem.2011.08.015 21871435 PMC3771870

[mgg3882-bib-0002] Betts, M. J. , & Russell, R. B. (2003). Amino acid properties and consequences of substitutions In Barnes M. R., & Gray I. C. (Eds.), Bioinformatics for Geneticists (pp. 289–316). Retrieved from http://onlinelibrary.wiley.com/doi/10.1002/0470867302.ch14/summary

[mgg3882-bib-0003] Brice, G. , Ostergaard, P. , Jeffery, S. , Gordon, K. , Mortimer, P. S. , & Mansour, S. (2013). A novel mutation in GJA1 causing oculodentodigital syndrome and primary lymphoedema in a three generation family. Clinical Genetics, 84(4), 378–381. 10.1111/cge.12158 23550541

[mgg3882-bib-0004] Choi, J. , Yang, A. , Song, A. , Lim, M. , Kim, J. , Jang, J.‐H. , … Jin, D.‐K. (2018). Oculodentodigital dysplasia with a novel mutation in GJA1 diagnosed by targeted gene panel sequencing: A case report and literature review. Annals of Clinical and Laboratory Science, 48(6), 776–781. 30610049

[mgg3882-bib-0005] De Bock, M. , Kerrebrouck, M. , Wang, N. , & Leybaert, L. (2013). Neurological manifestations of oculodentodigital dysplasia: A Cx43 channelopathy of the central nervous system? Frontiers in Pharmacology, 4, 10.3389/fphar.2013.00120 PMC3783840 24133447

[mgg3882-bib-0006] Hennekam, R. , Allanson, J. , & Krantz, I. (2010). Gorlin’s syndromes of the head and neck, 5th ed Oxford, NY: Oxford University Press.

[mgg3882-bib-0007] Jamsheer, A. , Sowińska‐Seidler, A. , Socha, M. , Stembalska, A. , Kiraly‐Borri, C. , & Latos‐Bieleńska, A. (2014). Three novel GJA1 missense substitutions resulting in oculo‐dento‐digital dysplasia (ODDD) ‐ further extension of the mutational spectrum. Gene, 539(1), 157–161. 10.1016/j.gene.2014.01.066 24508941

[mgg3882-bib-0008] Judisch, G. F. , Martin‐Casals, A. , Hanson, J. W. , & Olin, W. H. (1979). Oculodentodigital dysplasia. Four new reports and a literature review. Archives of Ophthalmology (Chicago, Ill.: 1960), 97(5), 878–884. 10.1001/archopht.1979.01020010436007 220941

[mgg3882-bib-0009] Kelly, S. C. , Ratajczak, P. , Keller, M. , Purcell, S. M. , Griffin, T. , & Richard, G. (2006). A novel GJA 1 mutation in oculo‐dento‐digital dysplasia with curly hair and hyperkeratosis. European Journal of Dermatology, 16(3), 241–245. 16709485

[mgg3882-bib-0010] Kjaer, K. W. , Hansen, L. , Eiberg, H. , Leicht, P. , Opitz, J. M. , & Tommerup, N. (2004). Novel Connexin 43 (GJA1) mutation causes oculo‐dento‐digital dysplasia with curly hair. American Journal of Medical Genetics. Part A, 127A(2), 152–157. 10.1002/ajmg.a.20614 15108203

[mgg3882-bib-0011] Kogame, T. , Dainichi, T. , Shimomura, Y. , Tanioka, M. , Kabashima, K. , & Miyachi, Y. (2014). Palmoplantar keratosis in oculodentodigital dysplasia with a GJA1 point mutation out of the C‐terminal region of connexin 43. The Journal of Dermatology, 41(12), 1095–1097. 10.1111/1346-8138.12682 25388818

[mgg3882-bib-0012] Kopanos, C. , Tsiolkas, V. , Kouris, A. , Chapple, C. E. , Albarca Aguilera, M. , Meyer, R. , & Massouras, A. (2019). VarSome: The human genomic variant search engine. Bioinformatics, 35(11), 10.1093/bioinformatics/bty897 PMC6546127 30376034

[mgg3882-bib-0013] Krutovskikh, V. , & Yamasaki, H. (2000). Connexin gene mutations in human genetic diseases. Mutation Research/Reviews in Mutation Research, 462(2–3), 197–207. 10.1016/S1383-5742(00)00037-5 10767631

[mgg3882-bib-0014] Laird, D. W. (2006). Life cycle of connexins in health and disease. Biochemical Journal, 394(3), 527–543. 10.1042/BJ20051922 16492141 PMC1383703

[mgg3882-bib-0015] Laird, D. W. (2014). Syndromic and non‐syndromic disease‐linked Cx43 mutations. FEBS Letters, 588(8), 1339–1348. 10.1016/j.febslet.2013.12.022 24434540

[mgg3882-bib-0016] Landrum, M. J. , Lee, J. M. , Benson, M. , Brown, G. R. , Chao, C. , Chitipiralla, S. , … Maglott, D. R. (2018). ClinVar: Improving access to variant interpretations and supporting evidence. Nucleic Acids Research, 46(D1), D1062–D1067. 10.1093/nar/gkx1153 29165669 PMC5753237

[mgg3882-bib-0017] Lee, J. R. , & White, T. W. (2009). Connexin‐26 mutations in deafness and skin disease. Expert Reviews in Molecular Medicine, 11, 10.1017/S1462399409001276 19939300

[mgg3882-bib-0018] MacArthur, M. W. , & Thornton, J. M. (1991). Influence of proline residues on protein conformation. Journal of Molecular Biology, 218(2), 397–412. 10.1016/0022-2836(91)90721-H 2010917

[mgg3882-bib-0019] Paznekas, W. A. , Boyadjiev, S. A. , Shapiro, R. E. , Daniels, O. , Wollnik, B. , Keegan, C. E. , … Jabs, E. W. (2003). Connexin 43 (GJA1) mutations cause the pleiotropic phenotype of oculodentodigital dysplasia. American Journal of Human Genetics, 72(2), 408–418. 10.1086/346090 12457340 PMC379233

[mgg3882-bib-0020] Paznekas, W. A. , Karczeski, B. , Vermeer, S. , Lowry, R. B. , Delatycki, M. , Laurence, F. , … Jabs, E. W. (2009). GJA1 mutations, variants, and connexin 43 dysfunction as it relates to the oculodentodigital dysplasia phenotype. Human Mutation, 30(5), 724–733. 10.1002/humu.20958 19338053 PMC13138855

[mgg3882-bib-0021] Pfenniger, A. , Wohlwend, A. , & Kwak, B. R. (2011). Mutations in connexin genes and disease. European Journal of Clinical Investigation, 41(1), 103–116. 10.1111/j.1365-2362.2010.02378.x 20840374

[mgg3882-bib-0022] Porntaveetus, T. , Srichomthong, C. , Ohazama, A. , Suphapeetiporn, K. , & Shotelersuk, V. (2017). A novel GJA1 mutation in oculodentodigital dysplasia with extensive loss of enamel. Oral Diseases, 23(6), 795–800. 10.1111/odi.12663 28258662

[mgg3882-bib-0023] Richardson, R. , Donnai, D. , Meire, F. , & Dixon, M. J. (2004). Expression of Gja1 correlates with the phenotype observed in oculodentodigital syndrome/type III syndactyly. Journal of Medical Genetics, 41(1), 60–67. 10.1136/jmg.2003.012005 14729836 PMC1757241

[mgg3882-bib-0024] Roscoe, W. , Veitch, G. I. L. , Gong, X.‐Q. , Pellegrino, E. , Bai, D. , McLachlan, E. , … Laird, D. W. (2005). Oculodentodigital dysplasia‐causing connexin43 mutants are non‐functional and exhibit dominant effects on wild‐type connexin43. The Journal of Biological Chemistry, 280(12), 11458–11466. 10.1074/jbc.M409564200 15644317

[mgg3882-bib-0025] Shao, Q. , Liu, Q. , Lorentz, R. , Gong, X.‐Q. , Bai, D. , Shaw, G. S. , & Laird, D. W. (2012). Structure and functional studies of N‐terminal Cx43 mutants linked to oculodentodigital dysplasia. Molecular Biology of the Cell, 23(17), 3312–3321. 10.1091/mbc.E12-02-0128 22809623 PMC3431933

[mgg3882-bib-0026] Snider, C. , Jayasinghe, S. , Hristova, K. , & White, S. H. (2009). MPEx: A tool for exploring membrane proteins. Protein Science: A Publication of the Protein Society, 18(12), 2624–2628. 10.1002/pro.256 19785006 PMC2821280

[mgg3882-bib-0027] Stenson, P. D. , Mort, M. , Ball, E. V. , Evans, K. , Hayden, M. , Heywood, S. , … Cooper, D. N. (2017). The human gene mutation database: Towards a comprehensive repository of inherited mutation data for medical research, genetic diagnosis and next‐generation sequencing studies. Human Genetics, 136(6), 665–677. 10.1007/s00439-017-1779-6 28349240 PMC5429360

[mgg3882-bib-0028] Tumminelli, G. , Di Donato, I. , Guida, V. , Rufa, A. , De Luca, A. , & Federico, A. (2016). Oculodentodigital dysplasia with massive brain calcification and a new mutation of GJA1 gene. Journal of Alzheimer’s Disease, 49(1), 27–30. 10.3233/JAD-150424 26444782

[mgg3882-bib-0029] Wiest, T. , Herrmann, O. , Stögbauer, F. , Grasshoff, U. , Enders, H. , Koch, M. J. , … Schwaninger, M. (2006). Clinical and genetic variability of oculodentodigital dysplasia. Clinical Genetics, 70(1), 71–72. 10.1111/j.1399-0004.2006.00631.x 16813608

[mgg3882-bib-0030] Yeager, M. , & Gilula, N. B. (1992). Membrane topology and quaternary structure of cardiac gap junction ion channels. Journal of Molecular Biology, 223(4), 929–948. 10.1016/0022-2836(92)90253-G 1371548

