# Supplementary material for: Two novel GJA1 variants in oculodentodigital dysplasia
Source: Mol Genet Genomic Med. 2019 Jul 25;7(9):e882. doi: 10.1002/mgg3.882 (PMC6732303; doi:10.1002/mgg3.882)
Supplement: Supplementary file 1 [file MGG3-7-e882-s001.docx]

**SUPPLEMENTARY DATA**

**Methodology**

***Editorial Policies and Ethical Considerations***

This study was conducted according to informed consent procedures as approved by the institutional ethics review board. Probands and their families gave their consent for participation in this study.

***Mutation detection***

The only coding exon (exon 2) of *GJA1* was amplified in two overlapping fragments of 925bp and 1079bp using primers described by Paznekas et al (Paznekas et al., 2003).

Briefly, 100 ng of DNA was amplified in 10 mM Tris-HCl pH 8.3, 50 mM KCl, 2,5 mM MgC_l2_, 50 µM dNTP, 0.05 µM primers and 2 UI of FastStart Taq DNA polymerase (Roche-Applied-Science) in a final volume of 25µl. Touch down thermocycling was carried out in a Biometra T3000 Thermocycler at 95°C for 6 minutes followed by 12 cycles at 95 °C for 10 seconds, 65 to 53°C (decrease of 1 °C per cycle) for 15 seconds , 72 °C for 30 seconds followed by 38 cycles at 95 °C for 10 seconds, 53°C for 15 seconds , 72 °C for 30 seconds and a final elongation step of 10 minutes at 72 °C. The quality and size of PCR products were verified on 1.5 % agarose gel. PCR products were purified on Microcon YM-100 (Millipore) and sequenced on both strands on ABI3130 with primers used for the PCR amplification using BigDye Terminator v3.1 Cycle Sequencing Kit (Applied Biosystems).

The detected variants were analyzed for pathogenicity according to established guidelines from the American College of Medical Genetics/Association for Molecular Pathology (ACMG/AMP). These guidelines standardize the process of variant classification by stratification into five categories (pathogenic, likely pathogenic, uncertain significance, likely benign, benign) based on a combination of computational, population, functional and segregation data (Richards et al., 2015). The functional impact and evolutionary conservation scores of identified non-synonymous amino acid substitutions was evaluated *in-silico* by a number of prediction tools including SIFT, PolyPhen2, FATHMM, FATHMM-MKL, MetaLR, MetaSVN , DANN, CADD, PROVEAN , MutationAssessor 1.0, MutationTaster 2 and LRT (Adzhubei et al., 2010; Dong et al., 2015; Kircher et al., 2014; Kumar, Henikoff, & Ng, 2009; Quang, Chen, & Xie, 2015; Schwarz, Cooper, Schuelke, & Seelow, 2014; Shihab et al., 2015)

**References**

Adzhubei, I. A., Schmidt, S., Peshkin, L., Ramensky, V. E., Gerasimova, A., Bork, P., … Sunyaev, S. R. (2010). A method and server for predicting damaging missense mutations. *Nature Methods*, *7*(4), 248–249. https://doi.org/10.1038/nmeth0410-248

Dong, C., Wei, P., Jian, X., Gibbs, R., Boerwinkle, E., Wang, K., & Liu, X. (2015). Comparison and integration of deleteriousness prediction methods for nonsynonymous SNVs in whole exome sequencing studies. *Human Molecular Genetics*, *24*(8), 2125–2137. https://doi.org/10.1093/hmg/ddu733

Kircher, M., Witten, D. M., Jain, P., O’Roak, B. J., Cooper, G. M., & Shendure, J. (2014). A general framework for estimating the relative pathogenicity of human genetic variants. *Nature Genetics*, *46*(3), 310–315. https://doi.org/10.1038/ng.2892

Kumar, P., Henikoff, S., & Ng, P. C. (2009). Predicting the effects of coding non-synonymous variants on protein function using the SIFT algorithm. *Nature Protocols*, *4*(7), 1073–1081. https://doi.org/10.1038/nprot.2009.86

Paznekas, W. A., Boyadjiev, S. A., Shapiro, R. E., Daniels, O., Wollnik, B., Keegan, C. E., … Jabs, E. W. (2003). Connexin 43 (GJA1) mutations cause the pleiotropic phenotype of oculodentodigital dysplasia. *American Journal of Human Genetics*, *72*(2), 408–418. https://doi.org/10.1086/346090

Quang, D., Chen, Y., & Xie, X. (2015). DANN: a deep learning approach for annotating the pathogenicity of genetic variants. *Bioinformatics (Oxford, England)*, *31*(5), 761–763. https://doi.org/10.1093/bioinformatics/btu703

Richards, S., Aziz, N., Bale, S., Bick, D., Das, S., Gastier-Foster, J., … ACMG Laboratory Quality Assurance Committee. (2015). Standards and guidelines for the interpretation of sequence variants: a joint consensus recommendation of the American College of Medical Genetics and Genomics and the Association for Molecular Pathology. *Genetics in Medicine: Official Journal of the American College of Medical Genetics*, *17*(5), 405–424. https://doi.org/10.1038/gim.2015.30

Schwarz, J. M., Cooper, D. N., Schuelke, M., & Seelow, D. (2014). MutationTaster2: mutation prediction for the deep-sequencing age. *Nature Methods*, *11*(4), 361–362. https://doi.org/10.1038/nmeth.2890

Shihab, H. A., Rogers, M. F., Gough, J., Mort, M., Cooper, D. N., Day, I. N. M., … Campbell, C. (2015). An integrative approach to predicting the functional effects of non-coding and coding sequence variation. *Bioinformatics (Oxford, England)*, *31*(10), 1536–1543. https://doi.org/10.1093/bioinformatics/btv009
